# Changes in physical function over time in rheumatoid arthritis patients: A cohort study

**DOI:** 10.1371/journal.pone.0280846

**Published:** 2023-01-23

**Authors:** Rafaela Cavalheiro do Espírito Santo, Joshua F. Baker, Leonardo Peterson dos Santos, Jordana Miranda de Souza Silva, Lidiane Isabel Filippin, Juliana Katarina Schoer Portes, Claiton Viegas Brenol, Rafael Mendonça da Silva Chakr, Ricardo Machado Xavier

**Affiliations:** 1 Laboratório de Doenças Autoimunes, Serviço de Reumatologia, Hospital de Clínicas de Porto Alegre, Santa Cecília, Porto Alegre, Brazil; 2 Post Graduate Program in Medicine: Medical Sciences, Federal Universidade Federal do Rio Grande do Sul, Porto Alegre, Brazil; 3 Philadelphia Veterans Affairs Medical Center and Perelman School of Medicine, University of Pennsylvania, Philadelphia, PA, United States of America; 4 Universidade La Salle, Canoas, RS, Brazil; Nippon Medical School, JAPAN

## Abstract

**Introduction:**

Self-reported disability is potentially influenced by many factors in patients with rheumatoid arthritis (RA). In this sense, we evaluated the association between self-reported disability and (1) clinical features, (2) muscle strength and (3) physical performance over time among patients with RA from two distinct patient cohorts.

**Materials and methods:**

Two independent prospective RA cohorts were analyzed. The Health Assessment Questionnaire (HAQ), Disease Activity Score in 28 Joints (DAS28), handgrip test, chair stand test, timed-up-and-go (TUG) test and Short Physical Performance Battery (SPPB) were performed at baseline and in follow-up. T test for independent samples, Mann-Whitney U test, Spearman correlation coefficients and linear regression with generalized estimating equations were performed to assess associations between individual constructs at baseline and over time.

**Results:**

A total of 205 total RA patients were included [North American Cohort (n = 115); Brazilian Cohort (n = 90)]. At enrollment, Brazilian men had better HAQ than North American men (p<0.001). Brazilian patients overall had lower muscle strength than North American patients (p<0.05). HAQ was associated with DAS28, handgrip test, chair stand test, TUG and SPPB (p<0.001) in both cohorts. Worsening of the DAS28 and chair stand test were each associated with worsening in HAQ in longitudinal analysis over time. Worsening of handgrip was also associated in with worsening HAQ in both cohorts (p<0.05). A worse TUG test was associated with worsening in HAQ in Brazilian cohort (p<0.05) and a worse SPPB was associated with worsening in HAQ in North American cohort (p<0.05).

**Conclusion:**

Greater disability measured by HAQ is closely associated with disease activity, pain, muscle strength, and physical performance among RA. Worsening in self-reported disability correlate with worsening clinical factors including objectively-observed physical function.

## Introduction

Rheumatoid arthritis (RA) is a systemic chronic autoimmune disease that affects articular synovial tissue and can lead to joint damage and deformities. Often, RA patients also present with sarcopenia: decreased muscle strength and muscle mass with significant impact on physical performance [1]. In addition to adverse changes to body composition, fat infiltration within the muscle is also observed in association with low physical function, low muscle strength and low physical activity [2].

Physical function is the ability to perform both basic and instrumental activities of daily living [3]. In RA patients, physical function is generally observed to be 24–34% lower than that observed for healthy controls [4]. We previously observed that most patients with RA demonstrate moderate deficits in self-reported physical function by the Health Assessment Questionnaire-Disability Index (HAQ-DI scores 1–2) [5]. With treatment, Andrade et al. [6] demonstrated that physical function improved after 9 years of follow-up, on average, as demonstrated by a reduction HAQ-DI from 1.4±0.05 to 0.9±0.1. However, loss of physical function persists among RA patients and studies have demonstrated that a high HAQ score early in the course of the disease is prognostic of a worse long-term outcome [7, 8]. The HAQ, being a subjective measure of physical function, is influenced by factors such as pain, which is not directly related to muscle deficits [9].

Cross-sectional studies have demonstrated that HAQ is negatively associated with muscle strength assessed by handgrip test and negatively associated with physical performance assessed by timed-up-and-go (TUG) test [10, 11]. Although improvement in the HAQ score may be observed during treatment of the inflammatory arthritis, the association between the self-reported HAQ scores and objective assessments of muscle strength or physical performance over time has been less well defined. Further, the identification and monitoring of sarcopenia, a risk factor for disability and loss of physical function in RA, would help to guide therapeutic and preventive approaches. We aimed to evaluate the association between self-reported physical function and clinical characteristics, muscle strength, and physical performance over time in RA patients.

## Materials and methods

### Study design

This prospective cohort study combined two independent cohorts of patients with RA. We conducted this study in accordance with STROBE guidelines [12].

### Settings

To evaluate associations between physical function, muscle strength and physical performance, we studied two independent cohorts of patients with RA. The institutional review boards of the University of Pennsylvania (registered under number #813724), Philadelphia VA Medical Center (registered under number #01427), United States America (USA) and Universidade Federal do Rio Grande do Sul (UFRGS), Hospital de Clínicas de Porto Alegre (HCPA), Brazil (registered under number 2015–0297) approved each respective study. The declaration of Helsinki principles were followed and all subjects gave written informed consent.

### Participants

#### Inclusion criteria

All participants were already under treatment at each institution. The patients were included if they agreed to participate and met the inclusion criteria. Therefore both samples represent a convenience sample.

#### University of Pennsylvania (North American) cohort (N = 115)

The Penn cohort was initiated in 2012 to evaluate alterations in body composition and bone structure in patients with RA. Subjects in the Penn cohort were recruited from the University of Pennsylvania Rheumatology practices and Philadelphia Veterans Affairs Medical Center and consisted of individuals aged 18–70 years old, diagnosed with RA according to the American College of Rheumatology/American College of Rheumatology/European League Against Rheumatism (ACR/EULAR) criteria [13] and with stage of functional classification of RA between 1–3.

#### Universidade Federal do Rio Grande do Sul (Brazilian) cohort (n = 90)

The Brazilian cohort was initiated in 2015 to evaluate alterations in body composition and physical function in patients with RA. Subjects in the Brazilian cohort were recruited from the Hospital de Clínicas de Porto Alegre (HCPA) and consisted of individuals aged 18–70 years old, and diagnosed with RA according to the American College of Rheumatology/American College of Rheumatology/European League Against Rheumatism (ACR/EULAR) criteria [13] and with stage of functional classification of RA between 1–3.

#### Exclusion criteria

Subjects with juvenile idiopathic arthritis (or another inflammatory arthritis), active cancer, a history of chronic diseases known to affect bone health (e.g. chronic kidney disease, liver disease, malabsorption syndromes), or pregnancy were excluded in both cohorts. In addition, patients with stage of functional classification of RA on 4, deformities in the lower limbs, and any surgical history in the previous year were also excluded.

### Measurements

Demographic and clinical data included age, gender, disease duration (years), presence of erosion and treatment regimen assessed by a review of medical records.

Disease activity was assessed by the Disease Activity Score-28 (DAS28). The DAS28 considers 28 tender and swollen joint counts, general health (GH; patient assessment of disease activity using a 100 mm visual analogue scale (VAS) with 0  =  best, 100  =  worst), plus levels of C-Reactive Protein (mg/liter)) [14].

Self-reported disability was assessed by the Health Assessment Questionnaire-Disability Index (HAQ-DI). The HAQ-DI comprises eight categories and the sum of scores is then divided by the number of categories, yielding a total score ranging from 0 (best) to 3 (worst) [15]. The RA patients were categorized in light disability (HAQ scores 0–1), moderate disability (HAQ scores 1–2) and severe disability (HAQ scores 2–3) [16].

Muscle strength was assessed by a handgrip test. The test was measured using a handheld dynamometer (Jamar Hydraulic Hand Dynamometer, Preston, USA [Brazilian cohort] or (Takei Scientific Instruments Co., Ltd., Japan [North American cohort]). Despite the differences between the equipment, their measurements may be considered equivalent [17]. The patient was instructed to squeeze the handle as hard as possible for 5 seconds, and the maximal isometric voluntary contraction (MIVC) of each hand was thus quantified. The measurement was repeated after a recovery period of 60 seconds, and the highest value of three MIVCs was considered for analysis. Handgrip strength values <27 kg for men and <16 kg for women were considered indicative of muscle weakness [1, 18]. For Brazilian patients in whom joint involvement impaired testing, adjustments were made to the handheld dynamometer as described elsewhere [19].

Chair stand test was performed as a measure of the strength of the lower limb muscles. This is a timed test where the participant is asked to stand up from a chair and sit back down as quickly as possible five times [20].

Physical Performance was assessed by the Timed-up-and-go (TUG) test in the Brazilian cohort and Short Physical Performance Battery (SPPB) in the American cohort. For the TUG test, individuals are asked to rise from a standard chair, walk to a marker 3 meters away, turn around, walk back and sit down again (Total of 6 meters). Any time ≥20 seconds was considered slow (low speed) [1, 21]. The SPPB is a widely used and simple test to measure lower extremity function through observed completion of tasks that mimic daily actions. Specifically, it examines an individual’s performance with regard to static balance, gait speed and timed chair-raises [1, 22].

### Statistical analysis

The Shapiro-Wilk method was used to test for normality to quantitative variables. The mean ± standard deviation (SD) was shown to parametric data and median (interquartile range) was shown to non- parametric data.

Cross-sectional analyses: The T test for independent samples performed to parametric data and Mann-Whitney U of independent samples test was performed to non-parametric data to compare groups (North American cohort and Brazilian Cohort). Spearman correlation coefficients were explored to assess univariate associations between self-reported disability (HAQ-DI) and DAS28-CRP, VAS of pain, muscle strength (handgrip test and chair stand test), and objective assessments of physical performance (TUG test and SPPB). Correlations were ranked as suggested by Dancey and Reidy [23]: r = 1.0 indicates perfect association; r = 0.7–0.9, strong association; r = 0.4–0.6, moderate association; r = 0.1–0.3, weak association; and r = 0, no association.

Linear regression with generalized estimating equations with Gamma distribution was performed to evaluate the associations between continuous measures of HAQ-DI and clinical assessments including physical function at baseline and evaluate associations between changes over time in each. The significance level was set at p ≤ 0.05 for all analyses. Statistical analyses were performed in PASW 18.0 Statistics for Windows.

## Results

### Participants and descriptive data

A total of the 205 RA patients were included in this study, 115 from the North American cohort and 90 from the Brazilian cohort. Most patients were women (53.0% in the North American cohort and 86.7% in the Brazilian Cohort). The mean age was 56.2 ± 10.5 years old in combined cohorts, without statistically significant difference between them, although American women were younger than Brazilian women (p = 0.027). The median (IQR) of VAS pain was 40.0 (15.0–60.5) mm in combined cohorts, without differences between the two cohorts (p = 0.72).

Most RA patients had low to moderate disease activity with a mean DAS28-C-Reactive Protein (CRP) of 3.1 ± 1.2. The Brazilian cohort had a greater presence of erosion than the North American group. On the other hand, the North American group had more tender joints, more swollen joints and higher CRP levels than the Brazilian cohort (p = 0.011, p = 0.002, and p<0.001, respectively), but there was no difference in patient global scores. American patients used less conventional synthetic disease-modifying antirheumatic drugs (csDMARDs) (p<0.001) and used more biologic (b) DMARDs (p = 0.003). Additional demographic and clinical details are described in Table 1.

**Table 1:** Basic characteristics of the two study cohorts.

	Combined cohorts (n = 205)	North American Cohort (n = 115)	Brazilian Cohort (n = 90)	P
Gender				
Women, n (%)	139 (67.8)	61.0 (53.0)	78 (86.7)	<0.001^3^
Men, n (%)	66 (32.2)	54.0.0(47.0)	12 (13.3)	
Age (years), mean±SD	56.2±10.5	56.0±12.5	56.5±7.2	0.750^1^
Women, mean±SD	55.2±10.1	53.1±12.8	56.9±7.0	0.027^1^
Men, mean±SD	58.2±11.0	59.2±11.3	53.4±8.3	0.052^1^
Disease duration (years), median (IQR)	8.3 (2.7–18.0)	8.3 (2.6–18.6)	8.5 (3.0–18.0)	0.683^2^
Presence of erosion, n (%)	93 (45.4)	29 (25.2)	64 (71.1)	<0.001^3^
DAS28-CRP, mean±SD	3.1±1.2	3.1±1.2	3.1±1.3	0.922^1^
VAS pain (mm), median (IQR)	40.0 (15.0–60.5)	40.0 (15.0–63.7)	41.0 (14.0–60.0)	0.722^2^
Patient global scores (mm), median (IQR)	39.0 (15.0–55.0)	35.0(15.0–50.0)	40.0 (10.0–70.0)	0.266
Remission, n (%)	54 (26.3)	29 (25.2)	25 (27.8)	0.846^3^
Low, n (%)	21 (10.2)	13 (11.3)	8 (8.9)	
Moderate, n (%)	79 (38.5)	48 (41.7)	31 (34.4)	
High, n (%)	44 (21.5)	25 (21.7)	19 (21.1)	
CRP mg/L	7.0 (5.0–12.0)	8.0 (5.0–13.0)	4.1 (2.0–9.9)	<0.001^2^
Treatment regimen				
Glucocorticoids, n (%)	96 (46.8)	51 (44.3)	45 (50.0)	0.481^3^
csDMARDs, n (%)	161 (78.5)	77 (67.0)	84 (93.3)	<0.001^3^
bDMARDs, n (%)	86 (42.0)	59 (51.3)	27 (30.0)	0.003^3^

p, values of the difference between the American group and Brazilian group

^1^ t test for independent samples

^2^ Mann-Whitney U test of independent samples

^3^Pearson’s chi-squared test; DAS-28–CRP, the Disease Activity Score-28 with C reactive protein; VAS pain, The Visual Analog Scale for Pain; CRP, C reactive protein; csDMARD, conventional synthetic disease-modifying antirheumatic drugs; bDMARDs, biologic disease-modifying antirheumatic drugs.

### Physical function, muscle strength and physical performance

At baseline, the combined median (IQR) of HAQ-DI scores was 0.8 (0.2–1.5). There was no statistically significant difference between North American and Brazilian women (0.8 (0.1–1.4) vs 1.0 (0.2–1.9) respectively, p = 0.153), while North American men had lower HAQ-DI scores than Brazilian men (0.6 (0.2–1.1) vs 1.8 (0.7–2.1) respectively, p<0.001) (Table 2).

**Table 2:** Descriptive data of physical function, muscle strength and physical performance on the two cohorts.

	Combined cohorts	North American Cohort	Brazilian Cohort	p
HAQ-DI scores	0.8 (0.2–1.5)	0.7 (0.2–1.3)	1.1 (0.3–1.9)	0.006^2^
Women	-	0.8 (0.1–1.4)	1,0 (0.2–1.8)	0.153^2^
Men	-	0.6 (0.2–1.1)	1.8(0.7–2.1)	<0.001^2^
Handgrip test				
Women (kg), mean±SD	16.9±9.0	19.8±9.2	14.7±8.3	0.013^1^
Men (kg), mean±SD	27.0±11.4	26.6±10.7	28.5±14.3	0.678^1^
Chair stand test				
Women (s), median (IQR)	15.1 (12.9–18.3)	10.3 (7.3–12.3)	15.5 (13.8–18.9)	<0.001^2^
Men (s), median (IQR)	11.4 (8.6–15.6)	11.5 (8.3–15.2)	14.6 (13.4–18.1)	0.007^2^
TUG	-	-	10.7 (9.4–13.0)	
Women (s), median (IQR)	-	-	10.9 (9.4–13.1)	
Men (s), median (IQR)	-	-	8.3 (7.3–11.1)	
SPPB	-	11.0 (9.0–12.0)	-	
Women (points), median (IQR)	-	12.0 (9.0–12.0)		
Men (points), median (IQR)	-	11.0 (9.0–12.0)		

p, values of the difference between the American group and Brazilian group

^1^ t test for independent samples

^2^ Mann-Whitney U test of independent samples. HAQ-DI, by the Health Assessment Questionnaire Disability Index; TUG test, Timed up and go test; SPPB, The Short Physical Performance Battery; kg, kilogram; IQR, Interquartile; SD, standard deviation.

The mean of muscle strength by handgrip test was 16.9 ± 9.0 kg for women and 27.0 ± 11.4 kg for men in the combined cohort. North American women had greater muscle strength compared to the Brazilian women (19.8 ± 9.2 kg vs 14.7 ± 8.3 kg, respectively; p = 0.013), while no statistically significant difference was found in muscle strength between North American and Brazilian men (Table 2; p = 0.678).

The median (IQR) of chair stand test was 15.1 (12.9–18.3) seconds for women and 11.4 (8.6–15.6) seconds for men. Again, North American women had shorter chair stand times suggesting better strength compared to the Brazilian women 10.3 (7.3–12.3) seconds vs 15.5 (13.8–18.9) seconds, respectively; p<0.001). North American men also had shorter chair stand times compared to the Brazilian men (11.5 (8.3–15.2) vs 14.6 (13.4–18.1); p = 0.007) (Table 2).

Physical performance was assessed by timed up and go (TUG) test in the Brazilian cohort and The Short Physical Performance Battery (SPPB) in the North American cohort. The median (IQR) of TUG was 10.7 (9.3–13.0) seconds. The women showed worse TUG performance than men (10.9 (9.4–13.1) vs 8.3 (7.3–11.1); p = 0.003). The median (IQR) of SPPB was 11.0 points in the North American cohort and was similar between women and men (Table 2; p = 0.618).

**Fig 1:**
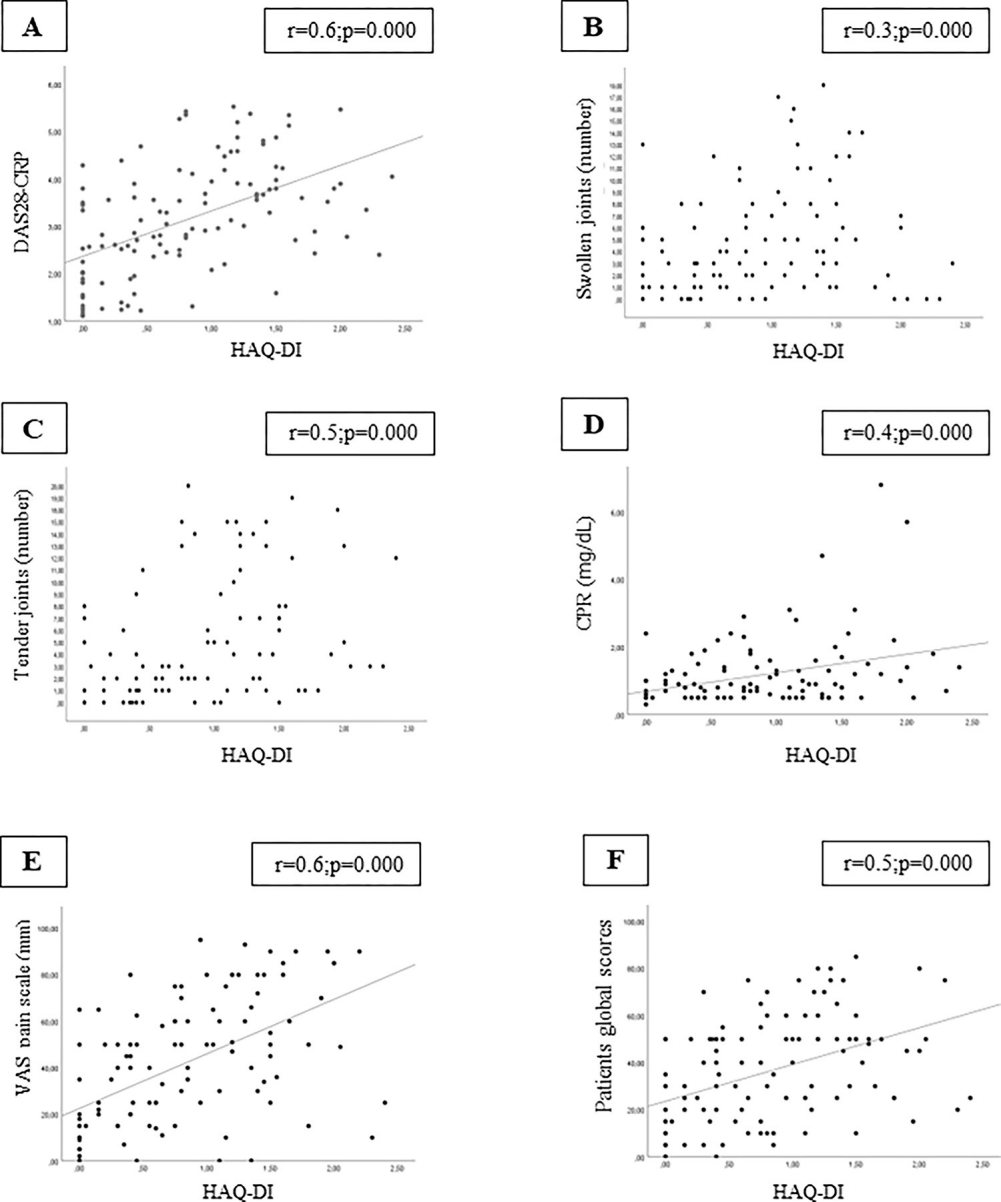
Correlation analysis was significant between HAQ-DI and (A) DAS28-CRP, (B) swollen joints. (C) Tender joints, (D) CRP, (E) VAS pain scale, and (F) patients global scores in the North American cohort. Abbreviations: HAQ-DI, Health Assessment Questionnaire-Disability Index; DAS28-CRP, Disease Activity Score in 28 Joints–C-reactive protein; VAS, visual analogue scale.

**Fig 2:**
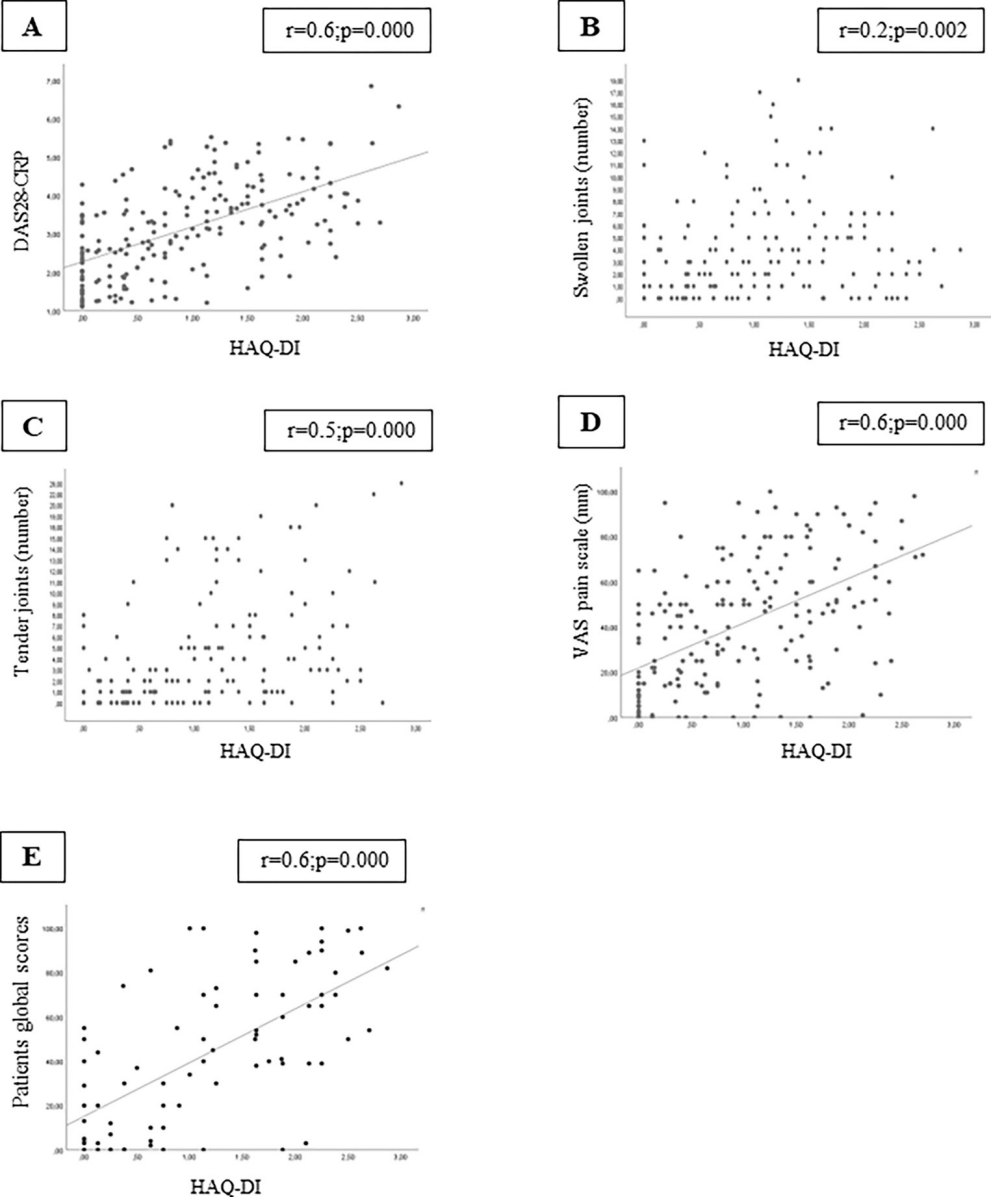
Correlation analysis was significant between HAQ-DI and (A) DAS28-CRP, (B) swollen joints. (C) Tender joints, (D) VAS pain scale, and (E) patients global scores in the Brazilian cohort. Abbreviations: HAQ-DI, Health Assessment Questionnaire-Disability Index; DAS28-CRP, Disease Activity Score in 28 Joints–C-reactive protein; VAS, visual analogue scale.

HAQ-DI scores were associated with DAS28, swollen joints, tender joints, CRP levels, VAS pain scale and patients global scores in the North American cohort (Fig 1; p<0.05). In the Brazilian cohort, HAQ-DI scores were associated with DAS28, swollen joints, tender joints, VAS pain scale and patients global scores (Fig 2; p<0.05). On the other hand, we did not find an association between HAQ-DI and disease duration in both cohorts (p>0.05).

**Fig 3:**
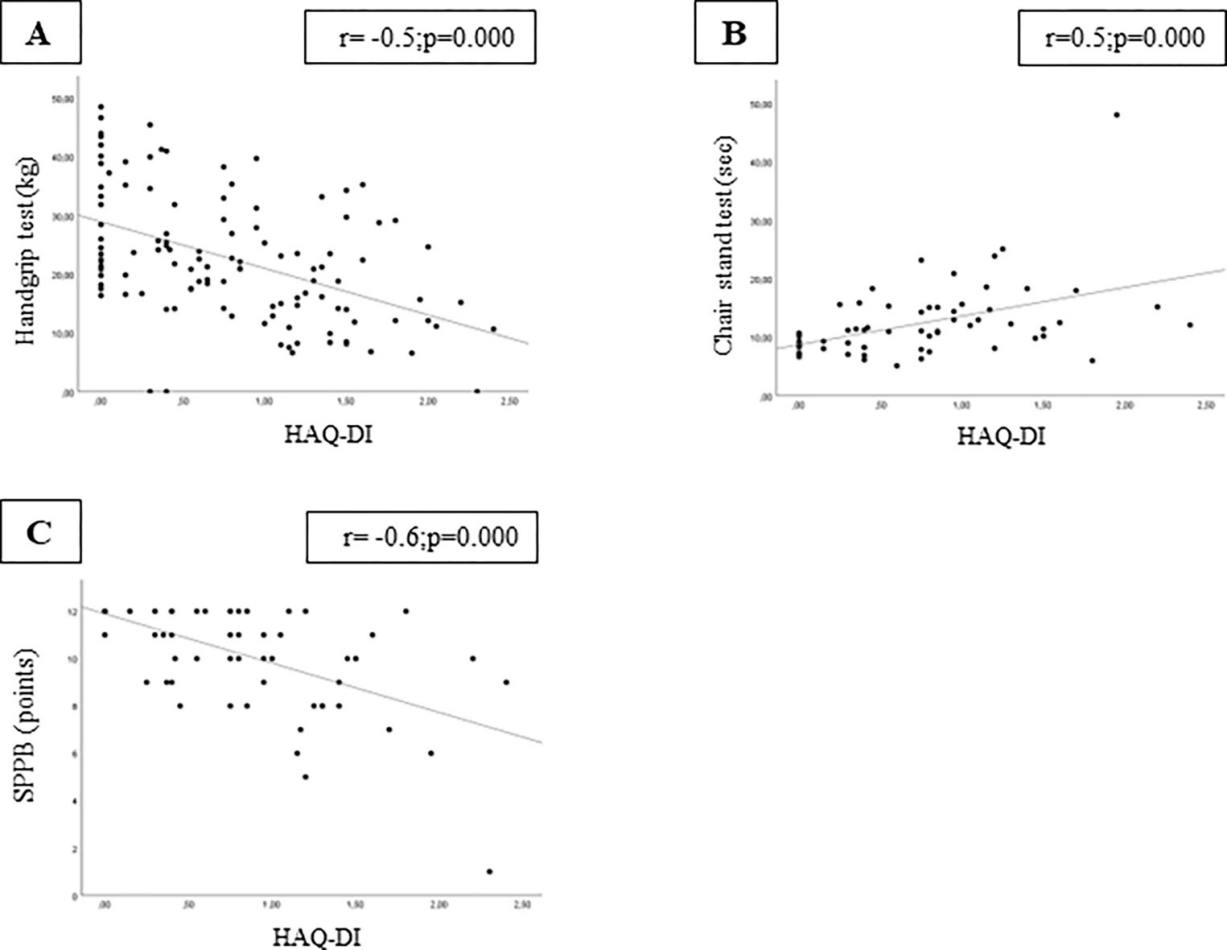
Correlation analysis was significant between HAQ-DI and (A) handgrip test, (B) chair stand test, and (C) SPPB in North American cohort. Abbreviations: SPPB, Short Physical Performance Battery; kg, kilogram; sec, seconds.

**Fig 4:**
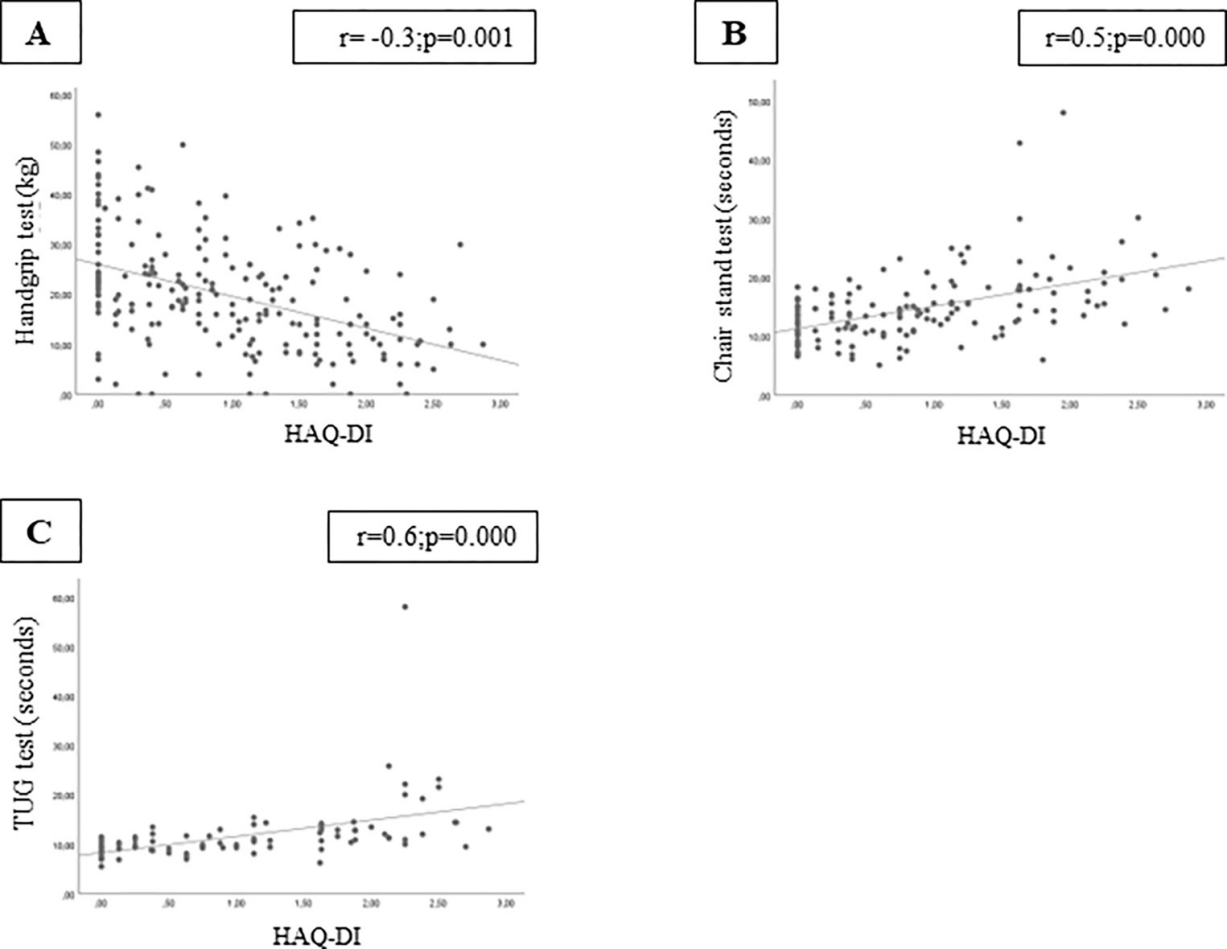
Correlation analysis was significant between HAQ-DI and (A) handgrip test, (B) chair stand test, and (C) TUG in the Brazilian cohort. Abbreviations: TUG, Timed-up-and-go test; kg, kilogram; sec, seconds.

HAQ-DI scores were associated with muscle strength by handgrip test and chair stand test in combined cohorts that were somewhat stronger in the American cohort (Figs 3 and 4). There was a strong positive association between the chair stand test and HAQ-DI in both cohorts. HAQ-DI scores were strongly associated with physical performance measured by both the TUG test and SPPB.

**Fig 5:**
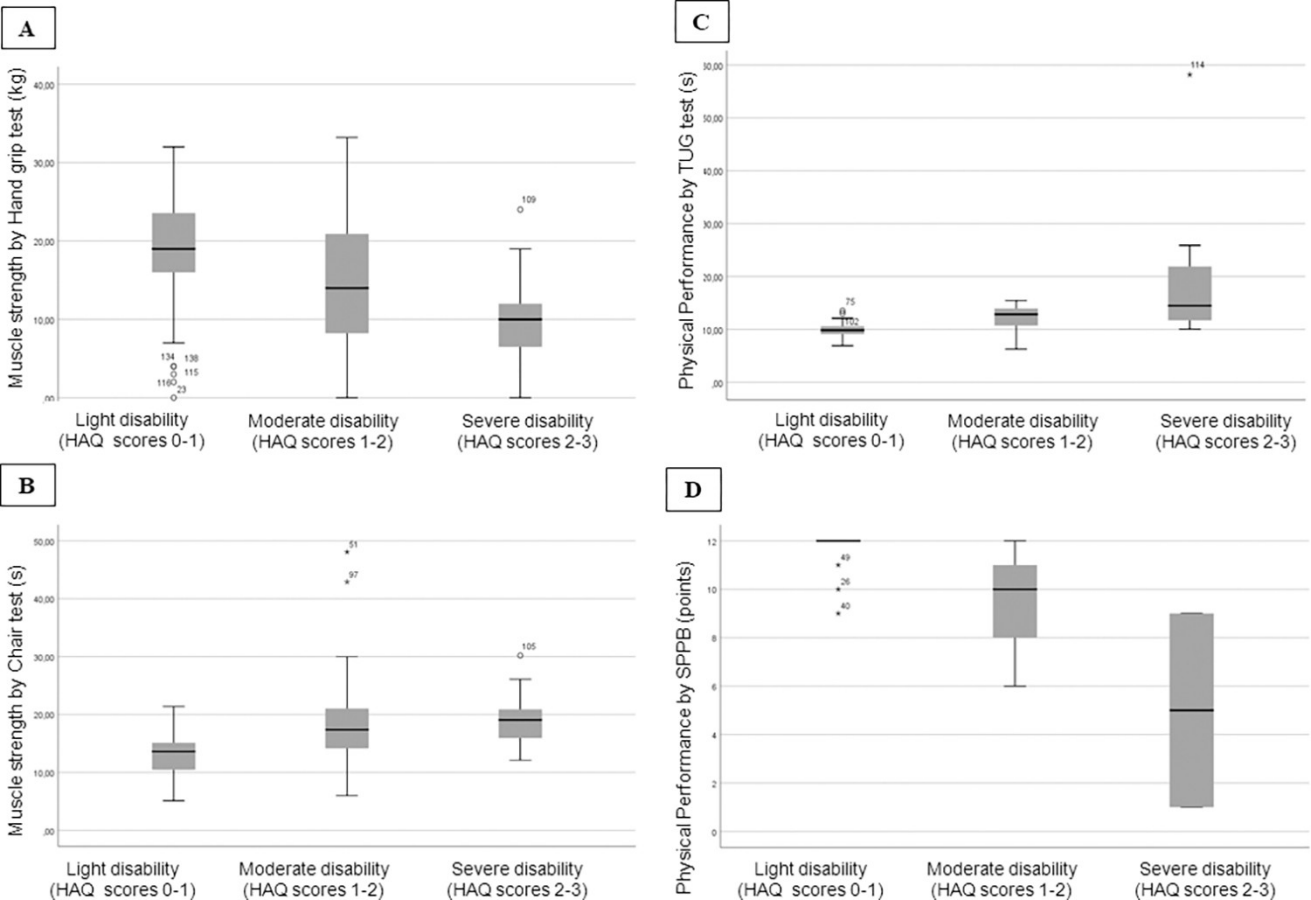
**A.** Mean and SD of muscle strength by handgrip test stratified by disability status of HAQ-DI. **B.** Median and (IQR) of muscle strength by chair test stratified by disability status of HAQ-DI. **C.** Median and (IQR) of physical performance by Timed up and go (TUG) test stratified by disability status of HAQ-DI. **D.** Median and (IQR) of physical performance by The Short Physical Performance (SPPB) test stratified by disability status of HAQ-DI. Independent Samples Kruskal-Wallis test for all analysis.

Women with lower HAQ-DI (HAQ-DI scores 0–1) demonstrated greater muscle strength by handgrip test (Fig 5A) and by chair stand test (Fig 5B), greater physical performance by TUG test (lower time of execution of TUG; Fig 5C; p<0.001), and greater physical performance by SPPB (higher SPPB score; Fig 5D; p = 0.004) compared to women with moderate (HAQ scores 1–2) and severe self-reported disability (HAQ scores 2–3). As few men reported severe disability, this analysis was not possible in this group.

Women and men in remission (DAS28-CRP <2.6) reported less disability by HAQ-DI and had greater muscle strength by handgrip test than those with high disease activity (DAS28 >5.1) (Table 3). Women in remission also showed greater muscle strength by chair stand test and greater physical performance by TUG test and SPPB (Table 3) than women with high disease activity. Among men, there was no significant association between disease activity and muscle strength by chair test or physical performance by SPPB. The analysis of the TUG was not possible in men because no men that performed the TUG test had high disease activity.

**Table 3:** Comparison of physical function, muscle strength and physical performance (disease remission versus high disease activity).

	Remission (n = 54)	High disease activity (n = 32)	p
*HAQ-DI scores*	0.2 (0.0–0.5)	1.4 (1.1–2.0)	0.003^1^
Women	0.1 (0.0–0.6)	1.6 (1.1–2.1)	<0.001^1^
Men	0.2 (0.0–0.6)	1.0(0.5–1.3)	0.003^1^
*Handgrip test*			
Women (kg), mean±SD	20.0±7.2	12.8±7.8	0.000^2^
Men (kg), mean±SD	36.2±13.1	25.1±9.0	0.012^2^
*Chair stand test*			
Women (s), median (IQR)	13.5 (10.5–16.3)	15.5 (13.0–18.3)	0.021^1^
Men (s), median (IQR)	11.2 (8.6–14.9)	12.5 (9–18.3)	0.331^1^
*TUG*			
Women (s), median (IQR)	9.4 (8.9–10.9)	13.1 (10.9–14.8)	<0.001^1^
Men (s), median (IQR)	7.5 (5.5–10.0)	#	-
*SPPB*			
Women (points), median (IQR)	12 (12–12)	9.5 (7.7–10.5)	0.008^1^
Men (points), median (IQR)	11 (10.0–12.0)	11 (8.0–12.0)	0.438^1^

54 patients in remission (29 from the United States x 25 from Brazil)—men 17 and women 37 / high activity: 12 men from the USA X 13 women from the USA and 19 women from Brazil = 32 patients with RA. p, values of the remission group and high disease activity group

^1^ Mann-Whitney U test of independent samples

^2^ t test for independent samples #Could not calculate. HAQ-DI, by the Health Assessment Questionnaire Disability Index; TUG test, Timed up and go test; SPPB, The Short Physical Performance Battery; SD, standard deviation; IQR, interquartile.

### Longitudinal associations between self-reported disability and muscle strength and physical performance

Over time, the North American cohort (24 months) and the Brazilian cohort (12 months) showed changes in muscle strength assessed by the handgrip test. On the other hand, only the Brazilian cohort showed alterations in the chair stand test. Lastly, only the Brazilian cohort showed changes in physical performance assessed by the TUG test (Table 4).

**Table 4:** Changes over time in self-reported physical function and clinical characteristics, muscle strength, and physical performance over time in the North American and Brazilian cohorts.

	North American Cohort		Brazilian Cohort	
	Baseline	At 24 months	Baseline	At 12 months
DAS-28-CRP, mean±SD	3.1±1.2	2.7±1.7	3.1±1.3	3.2±1.2
HAQ-DI scores, median (IQR)	0.7 (0.2–1.3)	0.6 (0.0–1.3)	1.1 (0.3–1.9)	1.0 (0.6–1.7)
Women, median (IQR)	0.8 (0.1–1.4)	0.6 (0.0–1.4)	1.0 (0.2–1.8)	1.0 (0.5–1.7)
Men, median (IQR)	0.6 (0.2–1.1)	0.6 (0.1–1.1)	1.8 (0.7–2.1)	0.7 (0.3–1.2)
Handgrip test				
Women (kg), mean±SD	19.8±9.2	16.9±7.6*	14.7±8.3	8.0±6.3*
Men (kg), mean±SD	26.6±10.7	29.5±11.3*	28.5±14.3	23.0±13.3*
Chair stand test				
Women (s), median (IQR)	10.3 (7.3–12.3)	10.3 (7.7–13.6)	15.5 (13.8–18.9)	14.5 (12.7–14.4)*
Men (s), median (IQR)	11.5 (8.3–15.2)	10.5 (7.4–13.1)	14.6 (13.4–18.1)	15.5 (13.8–18.9)*
TUG	-		10.7(9.4–13.0)	10.6(8.4–11.7)*
Women (s), median (IQR)	-	-	10.9(9.4–13.1)	9.9(8.6–12.0)*
Men (s), median (IQR)	-	-	8.3(7.3–11.1)	8.5(7.2–9.3)
SPPB	11.0 (9.0–12.0)	11.0 (9.7–12.0)	-	-
Women (points), median (IQR)	12.0 (9.0–12.0)	12.0 (9.0–12.0)		-
Men (points), median (IQR)	11.0 (9.0–12.0)	11.0 (10.0–12.0)		-

p, values of the difference over time on the American group and Brazilian group. DAS28-CRP, Disease Activity Score in 28 Joints–C-reactive protein; HAQ-DI, by the Health Assessment Questionnaire Disability Index; TUG test, Timed up and go test; SPPB, The Short Physical Performance Battery; kg, kilogram; IQR, Interquartile; SD, standard deviation

*, Statistically significant difference.

Changes in DAS28 over time were positively associated with changes in HAQ in both cohorts (p<0.05). Improvements in strength measured by handgrip and chair stand test were inversely associated with changes in HAQ in both the North American and Brazilian cohorts (p<0.05). Lastly, worsening performance by TUG test was positively associated with worsening in HAQ in the Brazilian cohort (p<0.05) and improved performance on SPPB was associated with improvements in HAQ in the North American cohort (p<0.05). These results are shown in Table 5.

**Table 5:** Evaluation of continuous variables those were associated with the HAQ-DI in longitudinal analyses. The beta-coefficient represents the relationship between the change in the variable and the change in HAQ-DI over the same follow-up time.

Variables	Cohorts	β	95%CI	p
Age (y)	BR	-0.018	-0.034–0.002	0.027
	US	-0.003	-0.013–0.006	0.513
Gender	BR	-0.297	-0.759–0.165	0.208
	US	0.170	-0.334–0.675	0.509
Disease duration	BR	0.015	0.001–0.030	0.042
	US	0.012	-0.001–0.025	0.063
DAS28-CRP	BR	0.202	0.140–0.265	<0.0001
	US	0.142	0.048–0.236	0.003
Handgrip test	BR	-0.013	-0.024–0.003	0.014
	US	-0.019	-0.032–0.006	0.004
Chair stand test	BR	0.019	0.010–0.028	<0.0001
	US	0.035	0.019–0.051	<0.0001
TUG test	BR	0.034	0.014–0.055	0.001
	US	-	-	-
SPPB	BR	-	-	-
	US	-0.128	-0.167–0.090	<0.0001

β, beta; 95%CI, 95% confidence intervals; y, years; DAS28, Disease Activity Score 28; CRP, C-reactive protein; TUG, timed-up and go; SPPB, Short Physical Performance Battery; BR, Brazil; US, United States.

## Discussion

The association between the changes in disability that are self-reported and objective assessments of muscle strength or physical performance over time has not been well studied in RA. RA patients are at greater risk of impaired physical function [4] over time, but the associations between self-report of disability and the components of sarcopenia are less clear. In our sample, self-reported disability was strongly associated with muscle strength and physical performance, and changes in disability were associated with changes in muscle strength and physical performance over long-term follow-up (>1 year; continuous analysis). Finally, we found that disease activity assessed by DAS28-CRP, muscle strength assessed by handgrip and chair stand tests, and physical function assessed by the TUG (Brazil) and SPPB (North American) tests were strongly associated with self-reported disability.

Evaluation of sarcopenia is currently composed of assessments of muscle strength, muscle mass and physical performance [1]. Previous studies have demonstrated that muscle mass itself is strongly associated with disability by HAQ-DI in RA patients [24–26]. In this study, we assessed disability by HAQ and its association with other components of sarcopenia such as muscle strength and found strong associations in cross-sectional analyses and in longitudinal analyses. Taken together, these findings support the HAQ as a helpful self-reported measure of disability that captures sarcopenia-related disability.

In our study, the North American cohort had higher CRP as well as more tender joints and swollen joints than the Brazilian cohort. Despite this, we did not find statistical differences in the DAS28-CRP or in the patient global scores between two cohorts. Interestingly, while the North American cohort showed high CRP levels and more tender joints and swollen joints, the American cohort showed less disability as measured by the HAQ-DI than the Brazilian cohort. In addition, American women showed greater muscle strength by handgrip and chair test than Brazilian women, while American men showed greater muscle strength by chair test than Brazilian men. These findings might be explained by a lower prevalence of sarcopenia in the North American RA patients [26].

In prior studies, patients with RA often have demonstrated low and moderate disability by HAQ [2, 4, 27, 28]. In a previous study by our group, treated RA patients showed deterioration of disability over time. In this study, we demonstrated that most patients had light and moderate disability by HAQ and few patients had important improvements in HAQ over time. Our findings may be explained by a lack of significant improvement in disease activity over time [29, 30].

In addition, we found associations between HAQ and direct measures of muscle strength (by handgrip and chair test) and physical performance (by TUG and SPPB). Corroborating our findings, Sferra et al. [10] demonstrated that HAQ showed negative correlation with handgrip test (right hand = r -0.585 and left hand = r -0.528; P<0.01). Douglas-Withers et al. [11] demonstrated that HAQ was moderately associated with handgrip strength (R^2^ = -0.285 [11] in RA patients. In addition, they showed that HAQ-DI was strongly associated with TUG (R^2^ of 0.671). Also, we found significant statistical differences in the mean of HAQ-DI between patients without presence of erosion and patients with presence of erosion in North American Cohort, where patients without presence of erosion showed light physical disability by HAQ-DI. On the other hand, patients with presence of erosion showed moderate physical disability by HAQ-DI. In addition, we found a mean difference of HAQ-DI in patients without the presence of erosion of -0.6 between the North American Cohort and Brazilian Cohort. Thus, we believe that higher frequency of erosion presence in Brazilian Cohort had influence on worse HAQ-DI. This finding corroborates with Gong et al. (2019) [31] where the authors found a modest negative linear correlation between Sharp and HAQ scores and longer disease duration (p< 0.001) in Chinese RA patients. In addition, in the REAL—Rheumatoid Arthritis in Real Life in Brazil [32], the authors demonstrated that the patients with erosion showed an HAQ-DI value higher than those without erosion. Therefore, from our findings, low muscle strength, worse physical performance and the presence of bone erosion seem to influence the HAQ score in patients with RA.

In addition, the HAQ-DI is a subjective tool and may be influenced by social, economic and cultural factors. It is known that sociodemographic, environmental and health conditions may influence physical function [33–35]. Furthermore, there is a higher prevalence of negative health self-perception among individuals exposed to stressful environments, with reduced sleeping time and little time spent on leisure activities (i.e. situations generally related to urban work) [36]. The Brazilian population has worse sociodemographic, environmental and health conditions than the North American population and we speculate that this difference may have influenced the HAQ-DI scores. Thus, while a correlation with disease indeed exists within both populations, there are a number of other factors that are likely to have led to overall higher scores in the Brazilian population including disease damage, sociodemographic, environmental factors, and other health conditions.

Our study is unique in that it is the first study to prospectively assess the association between the improvement of physical function and objective assessments of muscle strength or physical performance. When assessed as continuous measures, we observed that changes in disability were indeed associated with muscle strength and with objectively assessed physical function and disease activity.

This study has some limitations, such as the absence of a control group with healthy individuals matched by age and gender. Also, the addition of physical activity data over time could have enabled more sophisticated understanding of the impact of exercise. The small number of patients with very severe disability represented by HAQ made it difficult to study this severely-affected sub-group. Finally, the absence of data about the radiographic change of RA evaluated by such a Steinbrocker radiographic stage was a limitation. The relationship between the radiographic change of RA and HAQ-DI should be explored in further studies.

## Conclusion

In conclusion, greater self-reported disability measured by HAQ is closely associated with disease activity and pain, but also with muscle strength, and physical performance among RA patients both at baseline and over time. Changes in HAQ seem to be associated with changes in objectively assessed strength and physical function suggesting HAQ captures the patient impact of sarcopenia-related disability.
